# Perceptions of health and risk management among home care workers in Sweden

**DOI:** 10.1179/108331913X13746741513153

**Published:** 2013-10

**Authors:** A Larsson, L Karlqvist, M Westerberg, G Gard

**Affiliations:** 1 Division of Health and Rehabilitation, Department of Health Science, Luleå University of Technology, Sweden; 2 Division of Innovation and Design, Department of Business Administration, Technology and Social Sciences, Luleå University of Technology, Sweden

**Keywords:** Health, Risk management, Home care work

## Abstract

**Background:**

Municipal home care workers provide high-quality services to an increasing proportion of elderly people living in private homes. The work environments and working conditions of these workers vary to a great extent, implying rapid priority-making among both employers and employees to ensure that the work can be performed in a safe way.

**Objectives:**

This study aims to examine home care workers’ perceptions of health, risks, working conditions, and risk management within their organization.

**Method:**

The study was based on cross-sectional data collected from home care service staff in a municipality in the north of Sweden. Nursing assistants and care aides (*n* = 133) replied to a self-administered questionnaire. Descriptive statistics and between-group differences were analysed.

**Results:**

Home care work was perceived to require high levels of professional skill and ingenuity, a good psychosocial work situation, but required a high physical workload. The general health, the capacity and self-efficacy of the staff in relation to work were good. Difficulty in performing risk assessments and following safety regulations due to lack of time, equipment, and information were identified.

**Conclusion:**

There is a need to increase participation in risk assessments among the staff, improve management support, structures, and cooperation with other divisions of the social services and the medical care organizations.

## Introduction

Today, it is important to promote health and risk management within home-based service work; as this sector is rapidly changing, due to an aging population and financial limitations.^1^,^2^ Home based service work can be defined as jobs providing practical household chores, personal care and social support, and performing delegated medical tasks to elderly people (clients) living in private homes. Home care is seen as a cost-effective way of maintaining elderly people’s independence, but is also the mode of care preferred by many clients.^3^ In Sweden home care is provided through home help by the municipality. The policy in Sweden is based on the vision that elderly people should be supported to live in their home as long as possible. Allocation to home care services is guided by a set of criteria in several countries in Europe, for example the Nordic countries, Belgium and the Netherlands, and applied in a personal needs assessment procedure. Countries differ in the extent to which they have formalized the needs assessment. In Sweden the municipalities differ in risk assessment policies.^3^

Each home provides unique physical and social working conditions, so home care staff face a variety of health risks. Their work partly consists of routine tasks, but they must also cope with frequently changing conditions, such as new clients, changes in staff, attendance to acute situations, travelling and performing work alone.^1^,^2^,^4^ They suffer from a high frequency of work-related musculoskeletal disorders and injuries and have a low prevalence of sustainable work ability.^5^^–^7 Their lone work and highly variable conditions impose high demands on their abilities to make safe choices at work.^8^ Therefore, it is important to identify factors that can enhance safe working practices in their work. Increased attention must be paid to their wellbeing for the provision of a high-quality service and minimization of adverse events in service work.

Research of health and safety promotion shows the importance of focusing on positive factors at work and the potential resources that can be found there. The focus needs to be on both the process and the participation and ownership of those concerned.^9^,^10^ Job related conditions, e.g., structures and social support, need to be given increased attention as these aspects have a potential influence on employees’ capacity, participation, job control, and behaviour.^11^,^12^ Research has shown that many workers underestimate their actual risk of getting work-related musculoskeletal disorders.^13^ Perceived personal risk in a work situation can help to motivate the adoption of safer work behaviour. That is, to promote and support a greater control over the risk factors at work.^14^,^15^ Closely related to the process of increasing the control over decisions and actions, is the concept of ‘self-efficacy’, i.e., a belief in one’s own ability to overcome obstacles and bring about the results one requires.^16^ Self-efficacy is also a social construct. The perceived self-efficacy beliefs are influenced by the work task and by feedback on performance from other people in the work environment.^16^,^17^

The shared perceptions among members in a social unit of safety related policies and practices in an organization, will influence the workers’ perceptions of what kind of role behaviour is expected.^18^ Hence, a good safety climate can be considered as a positive work-related resource for actively encouraging employees to take their own health and safety into account in various situations at work.^19^ This is of particular interest in research concerning home care services, where both the process and participation in risk management are important. This study aims to describe perceptions of health, risks, working conditions, and risk management among home care staff in Sweden.

## Participants and Methods

### Participants

This study was set in a municipality in the north of Sweden. A total of 350 care aides and nursing assistants provide home care services to about 900 elderly people (clients). They work in different geographical areas, some in the centre of the town, and others in the countryside. They are managed by one head of home care services and 16 supervisors.

### Methods

#### A model for participatory risk management

**Figure 1:**
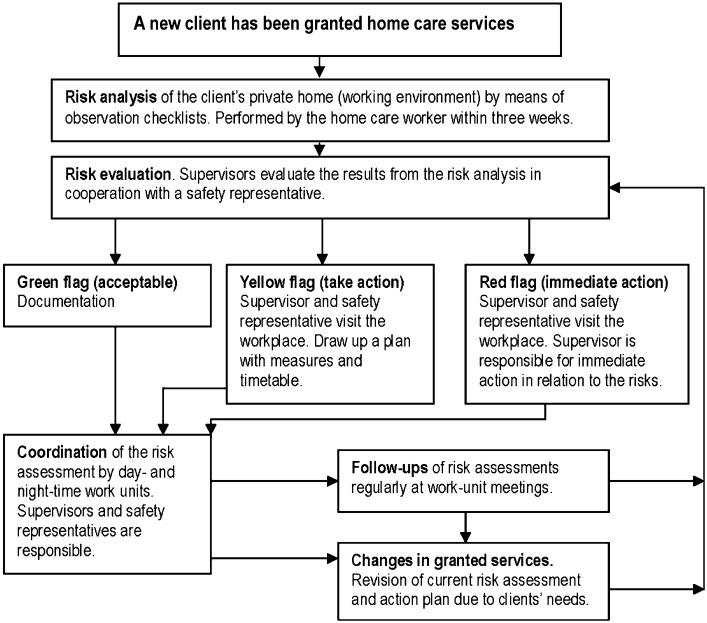
Process flow chart for the participatory risk management model.

The home care workers used a model for participatory risk management in their work in municipal home care services. The model was developed in 2006 by an internal workgroup in this municipality. The overall vision of the model is to support their self-management safety capacity and to enhance efficacy at identifying, documenting and managing risk factors relating to workers’ illnesses or accidents. The model consists of a process flow chart (Fig. 1) and supplementary checklists. The checklists (including aspects covering both the physical and psychosocial environment) enable a preparatory risk assessment to be performed by the home care staff in the home of each new client. All workplaces, about 900 private homes, are checked on a regular basis. Risk assessments are also performed for the general working environment (e.g., the staff room and the means of transport). This serves as a basis for the supervisor, by means of the process flow chart, to decide upon the measures that needs to be taken to address any environment related problems.

#### Procedure

This study was based on cross-sectional data gathered in February and March 2009 within a larger project for health and safety promotion in municipality-run home care services for the elderly. Initially, the researchers met with management of the home care services to plan the project, in addition to which, in the development of the questionnaire for data collection, one supervisor and two home care workers participated in meetings with the researchers. To ensure that the content of the questionnaire was perceived to be a relevant source of data, draft versions of the questionnaire were tested for face validity on representatives from the home care services staff.

The supervisors provided the researchers with lists of all the home care workers who met the criterion of having worked in the same home care unit for the last 6 months. This selection criterion was important for obtaining a representative value of the experiences of using the participative risk management model. As a result, 298 (out of a total of 350) home care workers were invited to participate in the study. The supervisors of these workers distributed a letter containing information, a letter of consent, a hard-copy questionnaire and a reply paid envelope in February 2009. A reminder was sent out after one month. The response rate varied in the different units and, overall, 158 (53%) of the home care workers agreed to participate. Of these, only 133 (45%) participants were included in the study since these participants completed all of the items on the questionnaire.

#### Data collection

Data were obtained through a comprehensive self-administrated questionnaire. The following variables and instruments were used:

1. Individual background factors: age, sex, profession, hours worked/week, seniority and work schedule were measured by items from the QPS Nordic-ADW^20^ modified to be relevant for the home care services setting.
2. Working conditions were measured using items derived from the Swedish version of the Job Content Questionnaire, graded on four or five point scales:^21^ Supervisor and co-worker support ‘when facing difficulties at work’ was measured by two single questions (scale end points 1 = ‘never’ and 5 = ‘always’). Two separate index variables measured the workers’ skill discretion on the requirements for skills and ingenuity required on the job (two items, scale end points 1 = ‘never’ and 4 = ‘often’) and decision-making authority, relating to what work to perform and how to perform it (two items). High levels of strain (‘yes’), defined as having high job demands and a low decision-making authority, were calculated. The index variable psychosocial job demands was produced by combining five items with the end points 1 = ‘never’ and 4 = ‘often’: the requirements to work fast and hard, needing to make a large amount of effort, having enough time to do the job and facing conflicting demands at work.^21^ The variable physical job demands (measuring the perceived physical exertion) was graded on the Borg RPE scale ranging from 6 to 20; end points ‘very, very low’ to ‘very, very high’.^22^ The general level of safety at work, e.g. requiring the respondent to make a general judgement about the safety in his/her own unit, was graded on a scale with the end points 1 = ‘very bad’ and 5 = ‘excellent’.^23^
3. Personal and participative risk management activities: A measure of participative safety behaviour aiming to measure the frequency with which the participants took part in risk management (assessed in terms of: never, sometimes or always) was developed within this study. There were eight questions on the perceived effects (yes/no) (e.g., assists with prioritization, co-operation) and one open-ended question to describe the pros and cons of the model. Compliance with personal protection regulations was measured using the Personal safety behaviour scale, with six items each measured on a seven-point scale (end points 1 = ‘never’ and 6 = ‘always’).^24^ The number of occasions when it was not possible to comply with safety regulations with regard to one’s own health and safety were measured with one single question using a five-point scale with the end points 1 = ‘never’ and 5 = ‘very often’, and one multiple-choice question and one open-ended question to describe the reasons.^25^ Self-efficacy in relation to work and safety was measured by an index variable produced by five items measured on a five-point scale with the end points 1 = ‘fully disagree’ and 5 = ‘fully agree’. The items reflected the respondent’s own capacity to handle most situations at work, to manage work as well as others, to have a positive view on work, to adjust work tasks to capacity^20^ and to influence safety at work.^25^
4. Health-related factors: On five-point scales, general health was estimated by one item with the end points 1 = ‘very poor’ and 5 = ‘very good’^26^ and psychological wellbeing by an index variable produced by three items on a scale ranging from 1 = ‘never’ to 5 = ‘often’.^27^ Musculoskeletal wellbeing during the previous month was measured in seven body areas (in upper part of back or neck; in lower back; in shoulders or arms; in hands or wrists; in hips; in knees; in feet or ankles) by seven items on a scale ranging from 1 = ‘every day’ to 5 = ‘very seldom or never’ experiencing pain.^28^ Ratings were calculated for each body area individually, and as an overall value (the ratings were summed and divided by seven to produce a variable ranging from one to five). The respondent’s work ability was measured by three items of the Work Ability Index, the first two being his or her work ability in relation to the physical and mental demands of the job (scale endpoints 1 = ‘very poor’ and 5 = ‘very good’), and the third being his or her belief about work ability in the present job two years from now (1 = ‘unlikely’, 4 = ‘not certain’, 7 = ‘yes, most likely’).^27^

### Data analysis

To test the scales (psychosocial job demands, decision-making authority, skill discretion, perceived self-efficacy, personal safety behaviour, psychological wellbeing, level of musculoskeletal wellbeing), principal component factor analyses and analyses of internal reliability were performed. After establishing that all scales were uni-dimensional through PCA, reliability was tested by calculating Cronbach’s alpha (Table 2). Doing this, two scales, each formed by two items, scored slightly under the preferred cut-off value of 0.70 but still higher than 0.60 that is seen as the lowest acceptable score. However, the vast majority of the scales showed levels above 0.7, indicating overall satisfactory reliability.^29^

**Table 1:** Characteristics of the participants included in the study (*n* = 133)

	%	Mean ±SD
Sex		
Women	92	
Men	8	
Age (years)		45.3±10.8
Position		
Care aide	43	
Assistant nurse	57	
Hours worked/week		34.4±4.8
Working full-time >37 hours/week	55	
Employment contract		
Permanent	93	
Temporary	7	
Work schedule		
Day, evening, weekend	94	
Night	6	
Seniority in home care service (years)		12.4±8.7
Time in present work unit (years)		8.8±7.5
Work unit size (number of co-workers)		26.1±11.1

**Table 2:** Results on working conditions, personal and participative risk management activities and health-related factors

	Overall study group (*n* = 133)			
	Score range	Alpha	Mean±SD	% ‘high’*
Working conditions				
Physical job demands	6–20	–	13.2±2.4	38
High levels of strain imposed by work (yes)	–	–		15
Psychosocial job demands	1–4	0.71	2.5±0.4	23
High decision-making authority	1–4	0.64	2.8±0.6	56
Skill discretion	1–4	0.62	3.6±0.5	96
Support from supervisor	1–5	–	3.7±0.9	64
Support from co-workers	1–5	–	3.9±0.9	74
General level of safety	1–5	–	3.2±0.6	29
				
Risk management activities				
Perceived self-efficacy	1–5	0.70	4.5±0.4	90
Participative safety behaviour:	1–3	–	–	23
Personal safety behaviour:	1–7	0.86	5.4±0.9	38
Occasions *not* possible to comply with safety regulations:	1–5	–	2.8±0.9	18
				
Health-related factors				
Level of general health	1–5	–	4.2±0.7	87
Psychological wellbeing	1–5	0.85	4.2±0.6	76
Level of musculoskeletal wellbeing	1–5	0.83	4.2±0.9	27
Correspondence work ability and physical job demands	1–5	–	4.3±0.7	88
Correspondence work ability and mental job demands	1–5	–	4.4±0.7	90
Positive belief about future ability to work	1, 4, 7	–	6.6±1.0	87

**Note:** *The cut-off points taken to describe ‘high’ levels of the aspects measured were:

Working conditions: ‘high’ physical job demands ≧14 ; strain = high psychosocial job demands and low decision-making authority ; ‘high’ psychosocial job demands, decision-making authority and skill discretion ≧3 ‘sometimes or often’; ‘high’ support ≧4 ‘most often or always’; and ‘high’ level of safety ≧4 ‘very god or excellent’.

Risk management activities: ‘high’ self-efficacy ≧4 ‘agree partially or fully; participative safety behaviour = 3 ‘always’; personal safety behaviour ≧6 ‘almost always or always’, and number of restricting occasions ≧4 ‘rather often or very often’.

Health-related factors: ‘high’ general health ≧ 4 ‘rather good’ or ‘very good’; ‘high’ psychological wellbeing ≧4 ‘quite often’ or ‘often’; ‘high’ musculoskeletal wellbeing = 5 ‘very seldom or never experiencing pain’; ‘high’ work ability in relation to, physical and mental, job demands ≧4 ‘rather good’ or’ very good’; and ‘high’ positive beliefs = 7 ‘yes, most likely’.

The mean, standard deviation and frequency measures were used to analyse the data. The cut-off points taken to describe ‘high’ levels of the aspects measured are given in the endnotes of the tables (Tables 2 and 3). The principle behind the selection of cut-off points was based on the significance of the response alternatives. For example, high physical job demand was defined as at, or above, 14 on the Borg RPE scale, which means perceiving the work to involve more than a ‘somewhat high’ physical exertion during an ‘ordinary’ working day. The differences between workers reporting ‘always’ and ‘never’ participating in the risk management in their work unit were analysed with ANOVA. The software programme SPSS version 17.0 was used, with a statistical significance of *P*<0.05.

**Table 3:** Results on differences between the workers’ reports of ‘always’ and ‘never’ participating in the risk management in their own work unit (only variables with statistically significant differences are presented in the table, with the relevant *P* values)

	Yes always (*n* = 30)			No, never (*n* = 13)			
		Mean±SD	% ‘high’*		Mean±SD	% ‘high’*	*P*†
Working conditions							
Decision-making authority (scale 1–4)		3.0±0.4	73		2.3±0.7	15	0.001
Support from supervisor (scale 1–5)		3.9±0.9	70		2.7±1.4	38	<0.001
Support from co-workers		4.1±0.9	80		3.3±1.4	46	0.032
General level of safety (scale 1–5)		3.4±0.6	37		2.8±0.8	15	0.006
							
Risk management activities							
Occasions not possible to comply with safety regulations: (scale 1–5)		2.5±0.8	10		3.3±1.3	23	0.018

**Note:** *The cut-off points taken to describe ‘high’ levels of the aspects measured were: ‘high’ decision-making authority ≧3 ‘sometimes or often’; ‘high’ support ≧4 ‘most often or always’; ‘ high level of safety ≧4 ‘very good or excellent’; ‘high’ number of restraining occasions ≧4 ‘rather often or very often’.

† The differences between the groups were analysed with ANOVA. Significance level <0.05.

### Ethics

The research was performed in compliance with the ethical principles of the Helsinki Declaration, and was approved by the Committee of Research Ethics at Umeå University, Sweden (Dnr 08-217 Ö).

## Results

### Study participants

The 133 participants whose responses were included had a mean age of 45 years, the majority were women, and 43% were care aides (Table 1).

### Working conditions

Nearly all, 96%, of the respondents considered their job to require high levels of professional skill and ingenuity, while 56% of them perceived themselves to have a high degree of decision-making authority (Table 2). Many of the respondents perceived themselves to have high levels of social support from their co-workers, but somewhat fewer perceived themselves to receive a high level of support from supervisors. A total of 37% respondents considered themselves to have high physical job demands, but less reported high psychosocial demands. The general level of safety at work was reported to be ‘acceptable’, with a median of 3.2 (Table 2).

### Personal and participative risk management activities

Having a high self-efficacy in relation to work and safety was perceived by 90% of the respondents. Self-reported safety behaviour was fairly high on average. However, 18% ‘rather often or often’ experienced conditions resulting in being unable to follow safety regulations (Table 2). The main reasons for this were a lack of time (stated by 50%), poor/inadequate equipment for household cleaning and a deficiency of ergonomic/lifting equipment (stated by 41%). Also, problems with shortage of staff, work scheduling, work routines and workload, cooperation within work units and psychosocial pressure from clients or their families/friends were mentioned. Some respondents specified that there were problems with timing; there was a ‘gap’ between the time when clients were discharged from the hospital and the time at which practical arrangements were resolved. The delay concerned the receipt of the required equipment and adequate information from other divisions of the social services and the medical care organizations.

In total, 23% of the respondents reported that they always participated in risk management in their unit (Table 2). The eight questions about perceived effects of the participative risk management model, received positive grades, from 49 to 66%, of the respondents. The highest agreements were given to the statements: ‘the model has contributed to improved agreement in my work unit regarding risk exposure in clients homes’ (66%) and ‘I have received sufficient training to be able to work safely’ (65%). The staff declared that the model has helped them to: develop good routines for improvements (57%), increase consensus of risk management in the client’s home (63%), and been a support in their work unit in priorities of interventions in clients’ homes (53%). Fifty-seven per cent perceived that they increased their insight in working environment questions, but about 50% perceived good cooperation with other divisions of the social service and with medical care about actions on decisions, and perceived that they had enough time and resources to perform the risk assessments in a good way. A lack of time to perform the assessments on a regular basis and insufficient follow-through in implementing changes were given as examples of reasons for not using the risk management model.

Differences between the individual respondents reports of ‘always’ participating in risk management in their unit and ‘never’ doing so were explored further (see Table 3). All variables (listed in Tables 1 and 2) were checked for significant differences between these two groups. The analysis showed that workers who always participated in participatory risk management perceived themselves to receive higher levels of social support from supervisors and co-workers, to have better decision-making authority, and to have a higher general level of safety at work. They also perceived conditions that restricted them from complying with safety regulations less often than those who ‘never’ participated in risk management (Table 3).

### Health-related factors

Having a high psychological wellbeing was reported by 75% of respondents and more than 87% perceived themselves to be in a good general state of health, to have good individual capacity in relation to the job demands and a positive belief in their future work ability. Musculoskeletal wellbeing, i.e., very seldom or never experiencing pain in any area of the body, was reported by 27% (Table 2). The neck, back, shoulders, and arms were the areas reported to be most commonly associated with experiences of pain.

## Discussion

### Discussion of the results

The respondents perceived themselves to be in good general health and to have good psychological wellbeing. Nearly all of our respondents perceived their job to require high skill levels and ingenuity, and many considered themselves to receive a considerable amount of social support and to have fairly good decision-making authority. These results are in line with earlier research, with the exception of the levels of health and psychological wellbeing that was higher in our study than in the literature.^6^,^30^ The work ability of the respondents was equal to that found in previous studies of female working populations.^31^ Despite this, the respondents reported musculoskeletal symptoms and physical demands. Environmental factors limited their ability to perform their work safely. This could imply that adverse events, physical exertion and pain are normalized and accepted by the home care staff. Even if a risk is recognized, a lack of necessary skills with which to manage it, unexpected situations, and the existence of psychosocial stress can hinder people from carrying out actions in the intended manner.^14^,^15^,^32^ Previous research showed that the physical workload predicts the risk of work-related musculoskeletal disorders.^33^ Proactive workplace interventions are needed for home care services staff to increase their wellbeing and sustain their present role in the long-term.

A high proportion of respondents perceived themselves as having high self-efficacy in relation to work and safety, with a mean value of 4.5 on a five-point scale. This is somewhat high in comparison with, for example, the mean value of 4.3 for teachers.^20^ Home care workers’ excellent ability to respond to clients’ needs has been described in previous research.^4^ However, perceived safety levels at work were reported to be only moderate in the results presented here. In general, safety was rated as 3.2 on a five-point scale. This is low when compared with the mean values of 3.4 in medical care and 3.8 in the petroleum industry, as reported previously.^23^ Self-efficacy is about what one can do with one’s skills in a specific situation.^8^,^16^ The respondents reported conditions that restricted their ability to comply with safety regulations and to participate in proactive risk management. In addition, their levels of physical exposure were high. Accordingly, there could be reasons for promoting their self-efficacy in managing front-line situations with regard to their own health and safety, despite these barriers. An increase in the actual opportunities for them to exert control over the conditions existing in their working environment is also needed.^19^,^34^,^35^

The model for participatory risk management aims to support the efficacy of each work unit in identifying and managing risk factors. Positive effects within the work units were reported by the staff, such as improved agreement regarding risk exposure and routines. This may have been a result of the use of observation checklists as the basis for discussions with peers and supervisors. However, the risk assessment model was not used in practice regularly. As few as 23% of the respondents reported that they ‘always’ participated in risk assessment at the arrival of new clients or when a change in health status or residence of existing clients occurred. Those who ‘always’ participated, reported stronger supervisory support in difficult situations at work, higher decision-making authority, a higher general level of safety and a low number of occasions when it was not possible to comply with safety regulations, compared to the workers who ‘never’ participated. These findings are supported by recent studies that showed the importance of structured routines;^7^ management commitment and support for changes in the workplace;^36^,^37^ and strengthening individual control over decisions and actions^19^,^35^ for workers’ health and safety. Research also shows differences between European countries in the allocation of responsibilities for home care policy, and in the use of risk assessment models. The policy on home care is often a national affair, while the organization and service provision is often decentralized. In Sweden, each employer is responsible for providing a safe working environment and the municipalities are responsible for providing home care to those in need of it.^3^

The low degree of participation in risk management and conditions restricting the respondents’ ability to make healthy and safe choices at work are a cause for concern. Earlier research showed that participative safety behaviour predicts the frequency of accidents within work groups.^38^ Hence, in a future revision of the existing risk management model, it is important to improve the preconditions for staff safe work practices, e.g., by ensuring that sufficient time is allowed and adequate information and equipment are provided. Coordination and communication with other divisions of the social services and with medical care organizations are needed earlier than at present. Risk assessment too, needs to be performed at an early stage. The home care services with their frequently changing conditions and need for coordination with other sectors have many features in common with highly dynamic medical care domains (for example rescue teams). Lessons learnt in the management of these sectors could be valuable for home care services, as research has confirmed that leadership, team work, and the teams’ safety-related behaviours had positive effects on the quality and safety of patient care as well as on the medical care staff’s wellbeing.^39^ There are indications that interventions which focus on potentially modifiable aspects of the safety climate can increase the health and safety of medical care personnel, as well as of patients.^23^,^40^

### Discussion of the method

This descriptive study is part of a health and safety project concerning municipality-based home care services. To attain the aim of this study we used a single point questionnaire survey with items or scales derived from standardized, reliable, and valid questionnaires, and a few additional questions were developed. The scales were tested and found to be reliable and valid for use in a home care context. Participation in the survey was voluntary and the response rate varied in the different work units. The mean age of those who declined to participate was significantly younger (median age 42) than that of the respondents, but these groups did not differ with regard to their overall age range, profession or sex. The known reasons for refusing to participate were lack of time and/or too extensive a questionnaire. It took about 10 minutes to complete the questionnaire. This implies that other factors may have influenced their willingness to participate, such as any possible lack of motivation and issues related to authority and dependencies. Those who perceived a high safety climate and commitment to good health and safety practices might have been more inclined to participate in the survey than other home care workers, which would imply a selection bias. The very high (and low) levels reported in some variables could, to some extent, have been influenced by biases, such as a recall bias, social expectations or protests. High levels might also be indicative of a ‘healthy worker effect’.^41^ The findings representing perceptions of one-third of the home care workers should therefore be interpreted with some caution.

However, this can be considered as an explorative study. Potentially modifiable aspects of the work setting were identified: areas in need of improvement as well as good practices. By using, and further building on these results in future practices and research, positive changes can be brought about for all employees within the home care services. Methods are often developed from a risk factor perspective, rather than a work health promotion perspective. More knowledge is needed of work conditions that promote health and work ability to improve home care workers’ working conditions.

### Conclusion

Home care work was perceived to require high levels of professional skill and ingenuity, a good psychosocial work situation, but implied a high physical workload. The general health, the capacity and self-efficacy of the staff in relation to work were good. Difficulties performing a risk assessment and following safety regulations due to lack of time, equipment and information were identified. There is a need to increase participation in risk assessments among the staff, improve management support, structures and cooperation with other divisions of the social services and the medical care organizations.
